# Bis[(dimethyl-λ^4^-sulfanyl­idene)oxonium] hexa­bromidotellurate(IV) dimethyl sulfoxide disolvate

**DOI:** 10.1107/S1600536808015468

**Published:** 2008-05-30

**Authors:** Martin D. Rudd, Gregory Kokke, Sergey V. Lindeman

**Affiliations:** a University of Wisconsin–Fox Valley, Menasha, Wisconsin 54952, USA; b Marquette University, Milwaukee, Wisconsin 53201, USA

## Abstract

The structure of the title salt, 2C_2_H_7_OS^+^·Br_6_Te^2−^·2C_2_H_6_OS, displays O—H⋯O hydrogen bonding between one protonated dimethyl sulfoxide mol­ecule and a neighboring dimethyl sulfoxide mol­ecule, and an octa­hedral geometry for the Te atom; the latter is situated on a center of inversion.

## Related literature

For the structure of the related compound [(dmso-H)_2_][TeCl_6_], see: Laitinen *et al.* (2002 ▶); Viossat *et al.* (1981 ▶). For related literature, see Abriel (1987 ▶); Abriel & du Bois (1989 ▶); Borgias *et al.* (1985 ▶); Jaswal *et al.* (1990 ▶); Keefer *et al.* (1988 ▶).
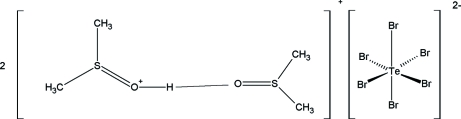


## Experimental

### 

#### Crystal data

2C_2_H_7_OS^+^·Br_6_Te^2−^·2C_2_H_6_OS
*M* *_r_* = 921.59
Triclinic, 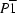

*a* = 8.0087 (2) Å
*b* = 9.2428 (2) Å
*c* = 10.5249 (3) Å
α = 66.280 (1)°
β = 70.732 (1)°
γ = 66.340 (1)°
*V* = 639.98 (3) Å^3^
*Z* = 1
Cu *K*α radiation
μ = 23.30 mm^−1^
*T* = 100 (2) K
0.23 × 0.20 × 0.16 mm

#### Data collection

Bruker APEX2 CCD detector diffractometer
Absorption correction: numerical [based on real shape of the crystal; absorption correction followed by the application of *SADABS* (Bruker, 2005 ▶)] *T*_min_ = 0.075, *T*_max_ = 0.118
5232 measured reflections
2112 independent reflections
2112 reflections with *I* > 2σ(*I*)
*R*_int_ = 0.023

#### Refinement

*R*[*F* ^2^ > 2σ(*F* ^2^)] = 0.021
*wR*(*F* ^2^) = 0.056
*S* = 1.15
2112 reflections
159 parameters
All H-atom parameters refined
Δρ_max_ = 1.02 e Å^−3^
Δρ_min_ = −0.68 e Å^−3^

### 

Data collection: *APEX2* (Bruker, 2005 ▶); cell refinement: *SAINT* (Bruker, 2005 ▶); data reduction: *SAINT*; program(s) used to solve structure: *XS* in *SHELXTL* (Sheldrick, 2008 ▶); program(s) used to refine structure: *SHELXL97* (Sheldrick, 2008 ▶); molecular graphics: *XP* (Bruker, 1998 ▶); software used to prepare material for publication: *XCIF* in *SHELXTL* (Sheldrick, 2008 ▶).

## Supplementary Material

Crystal structure: contains datablocks I, global. DOI: 10.1107/S1600536808015468/tk2262sup1.cif

Structure factors: contains datablocks I. DOI: 10.1107/S1600536808015468/tk2262Isup2.hkl

Additional supplementary materials:  crystallographic information; 3D view; checkCIF report

## Figures and Tables

**Table 1:** Hydrogen-bond geometry (Å, °)

*D*—H⋯*A*	*D*—H	H⋯*A*	*D*⋯*A*	*D*—H⋯*A*
O2—H2*O*⋯O1	0.83 (7)	1.62 (8)	2.448 (4)	175 (7)

**Fig. 1.**
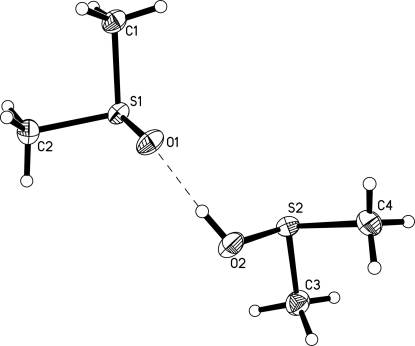
Numbering Scheme for [(DMSO)2H]+ (the hydrogen bond is shown as a dashed line). Displacement ellipsoids are shown at the 50% level.

**Fig. 2.**
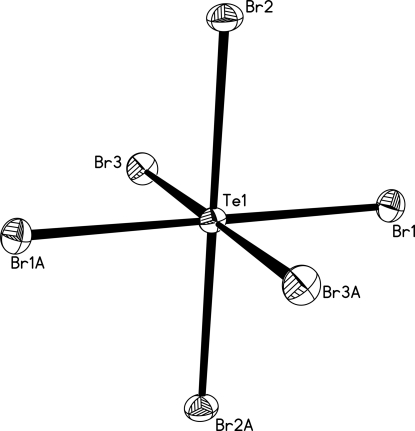
Numbering Scheme for [TeBr6]2-. Displacement ellipsoids are shown at the 50% level.

**Fig. 3.**
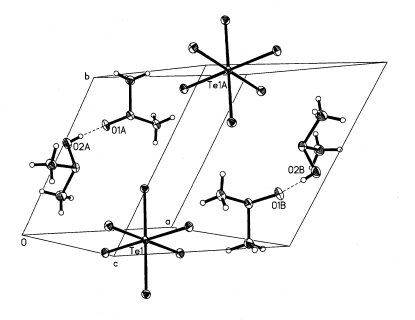
Hydrogen-bond formation and projection of the unit cell content of [(DMSO)2H]2[TeBr6]. Symmetry operators: Te1A [x, y+1, z]; O1A and O2A [1-x, 1-y, -z]; O1B and O2B [x, y, z+1]

## supplementary crystallographic information

### Comment

The structure of (I) consists of two units of two H^+^ hydrogen bonded dimethylsulfoxide molecules, Fig. 1, and a centrosymmetric hexabromotellurate(IV) anion, Fig. 2. At 2.448 (4) Å, the O1···O2 distance is relatively short, and is consistent with the presence of a moderately strong hydrogen bond (Keefer *et al.*, 1988). The IR spectrum reveals peaks typical for the [(dmso)_2_H]^+^ cation with a strong band at 731 cm^-1^. This is in line with similar samples in which the same cation has been analyzed (Jaswal *et al.*, 1990). A closely related tellurium complex, [(dmso)_2_H]_2_[TeCl_6_] has been structurally reported at room temperature (Viossat *et al.*, 1981) and at low temperature (Laitinen *et al.*, 2002). The cation in the latter experiment shows a O1···O2 distance of 2.435 (3) Å and the authors describe this as a "relatively strong hydrogen bond".

The hexabromotellurate(IV) anion in (I) shows an approximately octahedral geometry as expected. A review of some related structures shows that there are packing factors that slightly distort the geometry. One example where [TeBr_6_]^2-^ shows deviations away from the regular octahedral geometry indicates that there is a 0.024Å difference between the longest and shortest bond Te—Br bond lengths (Borgias *et al.*, 1985). In that report, the Te atom is located in a general position. In other literature, the Te is located at a center of inversion and displays a larger angular deviation from 90° [87.56 (3) - 92.44 (3)°] (Abriel & du Bois, 1989) which is greater than those reported here [less than 0.9° away from 90°]. A review of structural data for *MX*_6_E^2-^ compounds (*M* = Se, Te and *X* = Cl, Br, I) was published to provide an explanation of the stereochemistry of the lone pair electrons (Abriel, 1987).

The unit cell shows, Fig. 3, the pairs of hydrogen bonded dmso molecule and dmso-H ions and anions, Table 1.

### Experimental

Compound (I) was prepared by the slow cooling to room temperature of a hot solution (333 K) of tellurium dioxide (0.30 g, 0.19 mmol) dissolved in hydrobromic acid (1 mL) to which dimethylsulfoxide (5 mL) had been added. After 2 weeks, a crop of orange crystals formed although they are prone to solvent loss and decomposition. Analysis found: C 10.57; H 2.91; C_8_H_26_Br_6_O_4_S_4_Te requires: C 10.42, H 2.84. The IR spectrum showed strong bands at 3392, 1056, 731 cm^-1^.

### Refinement

The maximum and minimum electron density peaks of 1.01 and -0.68 e Å^-3^, respectively, are located 0.88 and 1.53 Å, respectively, from the Te atom. Hydrogen atoms positions were refined freely with C-H = 0.83 (7) - 1.03 (6) Å.

### Crystal data

**Table:**

2C_2_H_7_OS^+^·Br_6_Te^2–^·2C_2_H_6_OS	*Z* = 1
*M**_r_* = 921.59	*F*_000_ = 432
Triclinic, *P*1	*D*_x_ = 2.391 Mg m^−^^3^
Hall symbol: -P 1	Melting point: 343 K
*a* = 8.0087 (2) Å	Cu *K*α radiation λ = 1.54178 Å
*b* = 9.2428 (2) Å	Cell parameters from 4736 reflections
*c* = 10.5249 (3) Å	θ = 5–66º
α = 66.280 (1)º	µ = 23.30 mm^−^^1^
β = 70.732 (1)º	*T* = 100 (2) K
γ = 66.340 (1)º	Block, orange
*V* = 639.98 (3) Å^3^	0.23 × 0.20 × 0.16 mm

### Data collection

**Table:**

Bruker APEX2 CCD detector diffractometer	2112 independent reflections
Radiation source: fine-focus sealed tube	2112 reflections with *I* > 2σ(*I*)
Monochromator: graphite	*R*_int_ = 0.023
*T* = 100(2) K	θ_max_ = 66.8º
ω scans	θ_min_ = 4.7º
Absorption correction: numerical[based on real shape of the crystal; absorption correction followed by the application of SADABS (Bruker, 2005)]	*h* = −8→9
*T*_min_ = 0.075, *T*_max_ = 0.118	*k* = −9→10
5232 measured reflections	*l* = 0→12

### Refinement

**Table:**

Refinement on *F*^2^	Hydrogen site location: difference Fourier map
Least-squares matrix: full	All H-atom parameters refined
*R*[*F*^2^ > 2σ(*F*^2^)] = 0.021	*w* = 1/[σ^2^(*F*_o_^2^) + (0.0268*P*)^2^ + 1.1408*P*] where *P* = (*F*_o_^2^ + 2*F*_c_^2^)/3
*wR*(*F*^2^) = 0.056	(Δ/σ)_max_ = 0.004
*S* = 1.15	Δρ_max_ = 1.02 e Å^−^^3^
2112 reflections	Δρ_min_ = −0.68 e Å^−^^3^
159 parameters	Extinction correction: SHELXL97 (Sheldrick, 2008), Fc^*^=kFc[1+0.001xFc^2^λ^3^/sin(2θ)]^-1/4^
Primary atom site location: structure-invariant direct methods	Extinction coefficient: 0.00507 (17)
Secondary atom site location: difference Fourier map	

### Special details

**Table:**

Experimental. Analysis found: C 10.57; H 2.91; C~8~H~26~Br~6Õ~4~S~4~Te requires: C 10.42, H 2.84
Geometry. All e.s.d.'s (except the e.s.d. in the dihedral angle between two l.s. planes) are estimated using the full covariance matrix. The cell e.s.d.'s are taken into account individually in the estimation of e.s.d.'s in distances, angles and torsion angles; correlations between e.s.d.'s in cell parameters are only used when they are defined by crystal symmetry. An approximate (isotropic) treatment of cell e.s.d.'s is used for estimating e.s.d.'s involving l.s. planes.
Refinement. Refinement of *F*^2^ against ALL reflections. The weighted *R*-factor *wR* and goodness of fit *S* are based on *F*^2^, conventional *R*-factors *R* are based on *F*, with *F* set to zero for negative *F*^2^. The threshold expression of *F*^2^ > σ(*F*^2^) is used only for calculating *R*-factors(gt) *etc*. and is not relevant to the choice of reflections for refinement. *R*-factors based on *F*^2^ are statistically about twice as large as those based on *F*, and *R*- factors based on ALL data will be even larger.

### Fractional atomic coordinates and isotropic or equivalent isotropic displacement parameters (Å^2^)

**Table:**

	*x*	*y*	*z*	*U*_iso_*/*U*_eq_	
Te1	0.5000	0.0000	0.5000	0.01240 (12)	
Br1	0.47203 (5)	0.30103 (4)	0.30479 (4)	0.01875 (12)	
Br2	0.13998 (5)	0.11843 (5)	0.62389 (4)	0.01970 (12)	
Br3	0.39388 (5)	−0.09389 (5)	0.33507 (4)	0.02004 (12)	
S1	0.72250 (12)	0.24280 (11)	−0.11647 (9)	0.0176 (2)	
O1	0.8060 (4)	0.2997 (3)	−0.0395 (3)	0.0229 (6)	
C1	0.4776 (5)	0.3040 (5)	−0.0455 (4)	0.0213 (8)	
H1A	0.459 (7)	0.246 (6)	0.054 (5)	0.030 (12)*	
H1B	0.440 (7)	0.421 (7)	−0.066 (5)	0.032 (13)*	
H1C	0.424 (6)	0.263 (6)	−0.088 (5)	0.026 (12)*	
C2	0.7743 (6)	0.0237 (5)	−0.0301 (4)	0.0215 (8)	
H2A	0.700 (7)	−0.016 (6)	−0.060 (5)	0.027 (11)*	
H2B	0.741 (6)	0.002 (6)	0.070 (5)	0.027 (12)*	
H2C	0.907 (7)	−0.020 (6)	−0.066 (5)	0.025 (11)*	
S2	1.03170 (12)	0.54942 (12)	−0.30476 (10)	0.0219 (2)	
O2	1.0726 (4)	0.4039 (4)	−0.1636 (3)	0.0256 (6)	
H2O	0.985 (10)	0.364 (9)	−0.123 (8)	0.07 (2)*	
C3	1.2525 (6)	0.5183 (6)	−0.4190 (5)	0.0266 (9)	
H3A	1.340 (7)	0.519 (6)	−0.369 (5)	0.029 (12)*	
H3B	1.245 (7)	0.614 (7)	−0.496 (6)	0.040 (14)*	
H3C	1.284 (8)	0.410 (7)	−0.440 (6)	0.049 (15)*	
C4	1.0161 (7)	0.7251 (6)	−0.2660 (6)	0.0321 (10)	
H4A	1.015 (7)	0.822 (7)	−0.346 (6)	0.040 (14)*	
H4B	0.913 (8)	0.747 (7)	−0.207 (6)	0.043 (15)*	
H4C	1.125 (7)	0.695 (6)	−0.222 (5)	0.035 (13)*	

### Atomic displacement parameters (Å^2^)

**Table:**

	*U*^11^	*U*^22^	*U*^33^	*U*^12^	*U*^13^	*U*^23^
Te1	0.01221 (17)	0.01490 (18)	0.01250 (17)	−0.00478 (12)	−0.00205 (12)	−0.00641 (12)
Br1	0.0217 (2)	0.0180 (2)	0.0169 (2)	−0.00800 (15)	−0.00436 (15)	−0.00352 (15)
Br2	0.0143 (2)	0.0229 (2)	0.0215 (2)	−0.00556 (15)	0.00073 (15)	−0.01025 (16)
Br3	0.0237 (2)	0.0221 (2)	0.0211 (2)	−0.00733 (16)	−0.00826 (16)	−0.01003 (16)
S1	0.0187 (4)	0.0208 (4)	0.0158 (4)	−0.0096 (4)	−0.0020 (3)	−0.0059 (3)
O1	0.0228 (14)	0.0314 (15)	0.0234 (13)	−0.0161 (12)	−0.0018 (11)	−0.0118 (11)
C1	0.0215 (19)	0.026 (2)	0.022 (2)	−0.0115 (17)	−0.0024 (16)	−0.0095 (17)
C2	0.025 (2)	0.0208 (19)	0.021 (2)	−0.0087 (17)	−0.0077 (17)	−0.0047 (16)
S2	0.0167 (4)	0.0226 (5)	0.0279 (5)	−0.0070 (4)	−0.0058 (4)	−0.0075 (4)
O2	0.0243 (15)	0.0295 (15)	0.0261 (14)	−0.0158 (13)	−0.0052 (12)	−0.0042 (12)
C3	0.026 (2)	0.028 (2)	0.028 (2)	−0.0117 (18)	0.0002 (18)	−0.0107 (18)
C4	0.024 (2)	0.025 (2)	0.049 (3)	−0.0039 (18)	−0.002 (2)	−0.021 (2)

### Geometric parameters (Å, °)

**Table:**

Te1—Br1^i^	2.6865 (4)	C2—H2B	0.95 (5)
Te1—Br1	2.6865 (4)	C2—H2C	0.97 (5)
Te1—Br3	2.6956 (4)	S2—O2	1.576 (3)
Te1—Br3^i^	2.6956 (4)	S2—C3	1.767 (4)
Te1—Br2^i^	2.7103 (4)	S2—C4	1.776 (4)
Te1—Br2	2.7103 (4)	O2—H2O	0.83 (7)
S1—O1	1.537 (3)	C3—H3A	1.00 (5)
S1—C2	1.787 (4)	C3—H3B	0.92 (6)
S1—C1	1.791 (4)	C3—H3C	1.03 (6)
C1—H1A	0.96 (5)	C4—H4A	0.95 (6)
C1—H1B	0.95 (5)	C4—H4B	0.86 (6)
C1—H1C	0.97 (5)	C4—H4C	1.01 (5)
C2—H2A	0.98 (5)		
			
Br1^i^—Te1—Br1	180.0	H1B—C1—H1C	117 (4)
Br1^i^—Te1—Br3	89.604 (11)	S1—C2—H2A	107 (3)
Br1—Te1—Br3	90.395 (11)	S1—C2—H2B	110 (3)
Br1^i^—Te1—Br3^i^	90.397 (11)	H2A—C2—H2B	110 (4)
Br1—Te1—Br3^i^	89.604 (11)	S1—C2—H2C	103 (3)
Br3—Te1—Br3^i^	179.999 (1)	H2A—C2—H2C	112 (4)
Br1^i^—Te1—Br2^i^	89.151 (11)	H2B—C2—H2C	114 (4)
Br1—Te1—Br2^i^	90.849 (11)	O2—S2—C3	102.38 (19)
Br3—Te1—Br2^i^	89.485 (12)	O2—S2—C4	102.5 (2)
Br3^i^—Te1—Br2^i^	90.515 (11)	C3—S2—C4	100.2 (2)
Br1^i^—Te1—Br2	90.848 (11)	S2—O2—H2O	112 (5)
Br1—Te1—Br2	89.151 (11)	S2—C3—H3A	106 (3)
Br3—Te1—Br2	90.515 (11)	S2—C3—H3B	107 (3)
Br3^i^—Te1—Br2	89.485 (12)	H3A—C3—H3B	104 (4)
Br2^i^—Te1—Br2	180.0	S2—C3—H3C	107 (3)
O1—S1—C2	103.95 (17)	H3A—C3—H3C	115 (4)
O1—S1—C1	104.47 (17)	H3B—C3—H3C	116 (5)
C2—S1—C1	98.62 (19)	S2—C4—H4A	114 (3)
S1—C1—H1A	108 (3)	S2—C4—H4B	107 (4)
S1—C1—H1B	106 (3)	H4A—C4—H4B	108 (5)
H1A—C1—H1B	113 (4)	S2—C4—H4C	108 (3)
S1—C1—H1C	106 (3)	H4A—C4—H4C	110 (4)
H1A—C1—H1C	108 (4)	H4B—C4—H4C	111 (5)

Symmetry codes: (i) −*x*+1, −*y*, −*z*+1.

### Hydrogen-bond geometry (Å, °)

**Table:**

*D*—H···*A*	*D*—H	H···*A*	*D*···*A*	*D*—H···*A*
O2—H2O···O1	0.83 (7)	1.62 (8)	2.448 (4)	175 (7)

## References

[bb1] Abriel, W. (1987). *Z. Naturforsch. Teil B*, **43**, 415–420.

[bb2] Abriel, W. & du Bois, A. (1989). *Acta Cryst.* C**45**, 2002–2003.

[bb3] Borgias, B. A., Scarrow, R. C., Seidler, M. D. & Weiner, W. P. (1985). *Acta Cryst.* C**41**, 476–479.

[bb4] Bruker (1998). *XP* Bruker AXS Inc., Madison, Wisconsin, USA.

[bb5] Bruker (2005). *APEX2*, *SAINT* and *SADABS* Bruker AXS Inc., Madison, Wisconsin, USA.

[bb6] Jaswal, J. S., Rettig, S. J. & James, B. R. (1990). *Can. J. Chem.* **68**, 1808–1817.

[bb7] Keefer, L. J., Hrabie, J. A., Ohannesian, L., Flippen-Anderson, J. L. & George, C. (1988). *J. Am. Chem. Soc.* **110**, 3701–3708.

[bb8] Laitinen, R. S., Pietikäinen, J., Maaninen, A., Oilunkaniemi, R. & Valkonen, J. (2002). *Polyhedron*, **21**, 1089–1095.

[bb9] Sheldrick, G. M. (2008). *Acta Cryst.* A**64**, 112–122. 10.1107/S0108767307043930 18156677

[bb10] Viossat, B., Khodadad, P. & Rodier, N. (1981). *J. Mol. Struct.* **71**, 237–241.

